# Spermidine promotes nucleus pulposus autophagy as a protective mechanism against apoptosis and ameliorates disc degeneration

**DOI:** 10.1111/jcmm.13586

**Published:** 2018-03-25

**Authors:** Zengming Zheng, Zhou‐Guang Wang, Yu Chen, Jian Chen, Sinan Khor, Jiawei Li, Zili He, Qingqing Wang, Hongyu Zhang, Ke Xu, Gong Fanghua, Jian Xiao, Xiangyang Wang

**Affiliations:** ^1^ Department of Orthopaedic Surgery The Second Affiliated Hospital and Yuying Children's Hospital of Wenzhou Medical University Wenzhou China; ^2^ School of Pharmaceutical Sciences Key Laboratory of Biotechnology and Pharmaceutical Engineering Wenzhou Medical University Wenzhou China; ^3^ Department of Molecular Pharmacology Albert Einstein College of Medicine Bronx NY USA; ^4^ The Institute of Life Sciences Wenzhou University Wenzhou China

**Keywords:** apoptosis, autophagy, intervertebral disc degeneration, oxidative stress, spermidine

## Abstract

Spermidine has therapeutic effects in many diseases including as heart diastolic function, myopathic defects and neurodegenerative disorders via autophagy activation. Autophagy has been found to mitigate cell apoptosis in intervertebral disc degeneration (IDD). Accordingly, we theorize that spermidine may have beneficial effects on IDD via autophagy stimulation. In this study, spermidine's effect on IDD was evaluated in tert‐butyl hydroperoxide (TBHP)‐treated nucleus pulposus cells of SD rats in vitro as well as in a puncture‐induced rat IDD model. We found that autophagy was actuated by spermidine in nucleus pulposus cells. In addition, spermidine treatment weakened the apoptotic effects of TBHP in nucleus pulposus cells. Spermidine increased the expression of anabolic proteins including Collagen‐II and aggrecan and decreased the expression of catabolic proteins including MMP13 and Adamts‐5. Additionally, autophagy blockade using 3‐MA reversed the beneficial impact of spermidine against nucleus pulposus cell apoptosis. Autophagy was thus important for spermidine's therapeutic effect on IDD. Spermidine‐treated rats had an accentuated T2‐weighted signal and a diminished histological degenerative grade than vehicle‐treated rats, showing that spermidine inhibited intervertebral disc degeneration in vivo. Thus, spermidine protects nucleus pulposus cells against apoptosis through autophagy activation and improves disc, which may be beneficial for the treatment of IDD.

## 1 INTRODUCTION

Lower back pain (LBP) is a muscular complaint that can cause societal and economic problems in some instances due to intervertebral disc degeneration (IDD).^1^ Intervertebral disc degeneration (IDD) is intertwined with the process of ageing and occurs at a high incidence. It is one of the primary causes of neck pain, lower back pain and eventual disability, which significantly affects the quality of life and working ability of patients.^2^ Because the world's population is living longer, IDD is a large burden on public health and subsequent socio‐economic stability.^3^ IDD is commonly treated with bed rest, physiotherapy, pain relief and surgery. However, until recently, there have been no efficacious drugs for IDD therapy, giving rise to a demand to develop possible drug therapies.

The intervertebral disc is composed of three regions: the nucleus pulposus (NP), a highly hydrated core surrounded by a less hydrated, fibrous structure known as the annulus fibrosus (AF) and cartilaginous and bony endplates (EPs) at the boundaries with vertebral bodies.^4^ The nucleus pulposus is of vital importance to the physiological function and maintenance of intervertebral discs. Nucleus pulposus cells (NP cells) are the primary resident cells within the nucleus pulposus. They produce the main extracellular matrixes (ECM) components of the nucleus pulposus including Collagen‐II and aggrecan. These proteins aid in disc homoeostasis by blocking nerve ingrowth^5^ and disc calcification.^6^ Aberrant apoptosis within the nucleus pulposus is a key factor in the development of IDD and has been considered as a target for the treatment of disc degeneration.^7^, ^8^, ^9^

Autophagy is an essential catabolic process that contributes to intracellular quality control by clearing damaged organelles and proteins.^10^ Autophagy is closely associated with apoptosis and senescence in the pathogenesis of many diseases, including cancer,^11^ neurodegenerative diseases 12 and osteoarthritis.^13^ Therefore, it is essential to investigate the crosstalk between autophagy and apoptosis in NP cells during the pathogenesis of IDD.

Spermidine is defined as a cationic polyamine that exists in living cells and has been found to reduce the ageing process. Spermidine application to cell culture media or in vivo application to animals has been found to prolong lifespan in various model organisms similar to autophagy induction.^14^, ^15^ Oral supplementation of spermidine is capable of extending the lifespan of mice and exerts cardioprotective effects by reducing cardiac hypertrophy and preserving diastolic function in aged mice. These effects are mediated in part by control of autophagic flux.^16^ Recently, it was demonstrated that spermidine could stimulate autophagy in various tissues including the brain,^17^ eye^18^ and heart.^19^ We theorize that spermidine might exert protective effects on IDD through autophagy stimulation.

To test this, we induced oxidative stress with tert‐butyl hydroperoxide (TBHP), which is a common pathological mechanism of both apoptosis and senescence^20^ in NP cells. We further examined the therapeutic effects of spermidine on apoptosis in NP cells as well as in a puncture‐induced rat IDD model. Our research demonstrated that spermidine administration significantly increases NP cell autophagy and blocks apoptosis with low inherent toxicity. Ultimately, spermidine may be of potential value in the development of novel drugs for the treatment of IDD.

## 2 MATERIALS AND METHODS

### 2.1 Ethics statement

All surgical interventions, treatments and post‐operative animal care procedures were performed in strict accordance with the Animal Care and Use Committee of Wenzhou Medical University (No. 2015‐054).

### 2.2 Reagents and antibodies

Spermidine (S0266), 3‐methyladenine (M9281), TBHP (458139) and type II collagenases (1148090) were purchased from Sigma‐Aldrich (St Louis, MO, USA). p62 (ab56416), LC‐3(ab128025), Beclin‐1(ab6242), Atg7(ab52472), cleaved caspase‐3(ab32042), Bax(ab32503) and Bcl‐2(ab59348) antibodies were purchased from Abcam (Cambridge, UK). FITC‐labelled and horseradish peroxidase‐labelled secondary antibodies were purchased from Abcam (ab7086, ab191866, ab193651). 4′, 6‐diamidino‐2‐phenylindole (DAPI) was obtained from Beyotime (C1002, Shanghai, China).

### 2.3 Isolation and culture of NP cells

Forty Sprague‐Dawley rats (20 male and 20 female, 150‐200 g) were killed with an overdose of sodium pentobarbital. The spinal columns from L1 to L6 were removed carefully under aseptic conditions, and lumbar discs were collected. Gel‐like nucleus pulposus tissues were separated from lumbar discs, and the tissues were digested in 0.25% trypsin and 0.2% type II collagenase (Gibco) for approximately 3 hours at 37°C. Digested tissues were transferred as explants to DMEM (Gibco, Invitrogen, Grand Island, NY) with 10% foetal bovine serum (FBS; Hyclone, Thermo Scientific, Logan, UT, USA) and antibiotics (1% penicillin/streptomycin) and maintained at 37°C with 5% CO2. NP cells were moved out of the explants after 1 week. When confluent, the cells were harvested using 0.25% Trypsin‐EDTA (Gibco, Invitrogen). Next, cells were counted and replanted in 6‐well plates at the appropriate density. During passaging, no significant changes in the morphology of cells between primary cells (passage 0) and later passages (passage 2) were noticed.

### 2.4 Cell culture treatment

To induce apoptosis of nucleus pulposus cells, different concentrations of TBHP (50, 100, 200, 300 and 500 μmol/L) were added into the culture medium of nucleus pulposus cells for 24 hours. Cells were pre‐treated with different concentrations of spermidine (1, 5, 10, 20, 50, 100 and 200 μmol/L) for 24 hours before the addition of TBHP (100 μmol/L) where indicated. For autophagy experiments, 10 μmol/L 3‐methyladenine (3‐MA, an autophagy inhibitor) was used for 1 hour prior to spermidine administration. All experiments were performed in triplicate.

### 2.5 Cell viability

Cell viability was assayed with the cell counting kit‐8 (CCK‐8; Dojindo Co, Kumamoto, Japan) according to the manufacturer's instruction. Briefly, cells were planted in 96‐well plates (5000 cell/cm^2^) and incubated in DMEM with 10% FBS at 37°C for 24 hours. Cells were treated with TBHP, spermidine and 3‐MA as described above. Then, cells were washed with PBS; 10 μL of CCK‐8 dye was added to each well, and the plate was incubated for 1 hour. The absorbance of the wells was then measured at 450 nm by a microplate reader (Thermo, Rockford, IL, USA).

### 2.6 Transmission electron microscopy

NP cells were fixed in 2.5% glutaraldehyde overnight, then post‐fixed in 2% osmium tetroxide for 1 hour and stained with 2% uranyl acetate for 1 hour. After dehydration in an ascending series of acetone, samples were embedded into Araldite and cut into semithin sections, which were stained with toluidine blue. Sections were examined with a transmission electron microscope (Hitachi, Tokyo, Japan).

### 2.7 Western blot

Total cellular protein was isolated using RIPA with 1 mmol/L PMSF, and protein concentration measured using bicinchoninic acid reagents (Thermo, Rockford, IL, USA). 30 μg of protein was separated by sodium dodecyl sulphate‐polyacrylamide gel electrophoresis (SDS‐PAGE) and transferred to a polyvinylidene difluoride (PVDF) membrane (Bio‐Rad, USA). Membranes were blocked with 5% non‐fat milk and incubated with primary antibodies at the indicated concentrations overnight at 4°C: cleaved caspase‐3 (1:1000), Bax (1:1000), Bcl‐2 (1:1000), Collagen‐II(1:1000), Aggrecan(1:1000), MMP13(1:1000), Adamts‐5(1:1000), Beclin‐1 (1:1000), LC3 (1:500), p62 (1:1000), Atg7 (1:1000), GAPDH(1:10 000) followed by the respective secondary antibodies. Blots were developed with ECL plus reagent (Invitrogen) and quantified using Image Lab 3.0 software (Bio‐Rad).

### 2.8 Immunofluorescence

LC3 (Abcam, Cambridge, UK), MMP13 (Abcam, Cambridge, UK) and Collagen‐II (Abcam, Cambridge, UK) staining were performed following 4% paraformaldehyde fixation and incubated with Triton X‐100 for 10 minutes. After blocking with 5% bovine serum albumin for 30 minutes, slides were incubated with primary antibodies against LC3 (1:200), MMP13 (1:200) or Collagen‐II (1:100) overnight at 4°C. The following day, samples were washed and incubated with FITC‐ or TRITC‐conjugated second antibodies for 1 hour and labelled with DAPI for 5 minutes. Finally, three fields of each slide were chosen randomly for observation with a fluorescence microscope (Olympus Inc., Tokyo, Japan).

### 2.9 TUNEL method

NP cells were collected after 12 hours in a six‐well plate. After fixing with 4% paraformaldehyde for 1 hours, cells were incubated with 0.1% Triton X‐100 for 10 mins and washed with PBS three times. According to the manufacturer's instructions, cells were stained with in situ cell death detection kit (F. Hoffmann‐La Roche Ltd., Basel, Switzerland) and 40,6‐diamidino‐2‐phenylindole (DAPI). Apoptotic changes were measured using a fluorescence microscope (Olympus Inc., Tokyo, Japan).

### 2.10 Surgical procedure

Rats were weighed and given an intraperitoneal (IP) injection of 2% (w/v) pentobarbital (40 mg/kg). As described previously,^21^ the experimental level rat tail disc (Co7/8) was located by digital palpation on the coccygeal vertebrae and confirmed by counting the vertebrae from the sacral region in a trial radiograph. Needles (27G) were used to puncture the whole layer of annulus fibrosus through the tail skin. To make sure the needle did not puncture too deep, the needle length was decided according to the annulus fibrosus and the nucleus pulposus dimensions, which were measured in the preliminary experiment and found to be about 4 mm. All the needles were kept in the disc for 1 minutes. Spermidine was diluted with normal saline and injected intraperitoneally after surgery at a dose of 50 mg/kg per day until the rats were killed. Daily monitoring of the rats was carried out to ensure their well‐being, and all animals were allowed free unrestricted weight bearing and activity.

### 2.11 Magnetic resonance imaging method

After 8 or 16 weeks of puncture, animals were given an MRI examination. Magnetic resonance imaging was performed on all rats to evaluate the signal and structural changes in sagittal T2‐weighted images using a 3.0 T clinical magnet (Philips Intera Achieva 3.0MR). T2‐weighted sections in the sagittal plane were obtained in the following settings: fast‐spin echo sequence with time to repetition (TR) of 5400 ms and time to echo (TE) of 920 ms; 320 (h) 9 256 (v) matrix; field of view of 260; and four excitations. The section thickness was 2 mm with a 0‐mm gap. The MRIs were evaluated by a blinded orthopaedic researcher using the classification system for intervertebral disc degeneration as reported by Pfirrmann et al^22^ (1 point = Grade I, 2 points = Grade II, 3 points = Grade III, 4 points = Grade IV).

### 2.12 Histopathologic analysis

Rats were killed by an IP overdose injection of 10% chloral hydrate, and the tails were harvested 8 and 16 weeks after surgery. The specimens were decalcified and fixed in formaldehyde, dehydrated and embedded in paraffin. Tissues were cut into 5‐μm sections. Slides of each disc were stained with safranin O‐fast green (S‐O). The cellular density and morphology of the nucleus pulposus and annulus fibrosus were examined by blinded researchers using a microscope and evaluated using a grading scale as described previously.^21^ The histologic score was 5 for a normal disc, 6‐11 for a moderately degenerated disc and 12‐14 for a severely degenerated disc.

### 2.13 Statistical analysis

All experiments were performed at least three times. All data are expressed as mean ± SEM. The statistical analyses were performed by one‐way analysis of variance (ANOVA) followed by Dunnett's multiple comparison test. A probability level of *P* < .05 was set as statistically significant. Each experiment consisted of at least three replicates per condition.

## 3 RESULTS

### 3.1 Spermidine treatment inhibits apoptosis in nucleus pulposus cells

**Figure 1:**
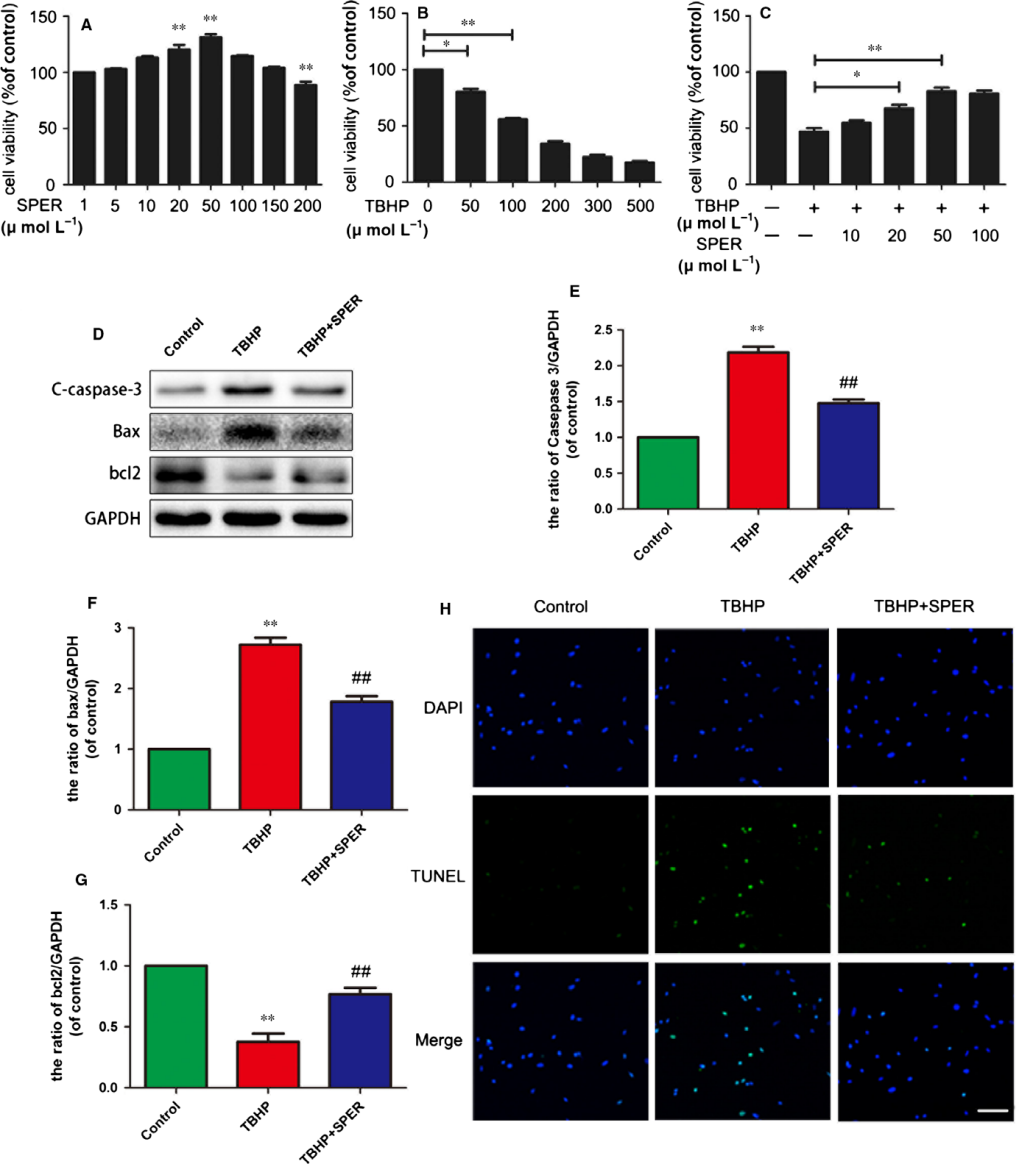
Spermidine treatment inhibits TBHP‐induced nucleus pulposus cell apoptosis. Nucleus pulposus cells were untreated, or treated with TBHP alone, or treated with spermidine (50 μmol/L) and TBHP, or treated with TBHP (A) Cell Counting Kit‐8 (CCK‐8) results of nucleus pulposus cells treated with different concentrations of spermidine for 24 hours. (B) CCK‐8 results of nucleus pulposus cells treated with different concentrations of TBHP for 4 hours. (C) CCK‐8 results of spermidine pre‐treated nucleus pulposus cells induced by TBHP. (D‐G) the protein expression of c‐caspase3, Bax, Bcl‐2 of the nucleus pulposus cells as treated above. (H) TUNEL assay was performed in nucleus pulposus cells as treated above (original magnification × 200, scale bar: 50 μm). The data in the figures represent the averages ± SD. *P < .05, **P < .01 vs the control group. ^##^ *P* < .01 vs TBHP group, n = 3 per group

Spermidine was not shown to be cytotoxic to nucleus pulposus cells after 24 hours at concentrations <100 μmol/L (Figure 1A). However, cell viability was found to be decreased with TBHP treatment in a dose‐dependent manner. Spermidine was shown to exhibit protective effects against TBHP‐induced cellular mortality (Figure 1B and C). Furthermore, TBHP (100 μmol/L) treatment markedly decreased the amount of anti‐apoptotic Bcl‐2 (*P* < .01), while increasing the amount of pro‐apoptotic Bax and cleaved caspase‐3 (*P* < 0.01). Pre‐treatment with spermidine markedly attenuated the increased protein levels of Bax and cleaved caspase‐3, while decreasing Bcl‐2 compared to the untreated group (*P* < .01; Figure 1D‐G). The TUNEL assay results showed that apoptotic incidence was markedly increased in cells treated with TBHP alone and reduced in spermidine‐treated cells (Figure 1H).

### 3.2 Spermidine regulates the expression of anabolic‐ and catabolic‐related proteins

**Figure 2:**
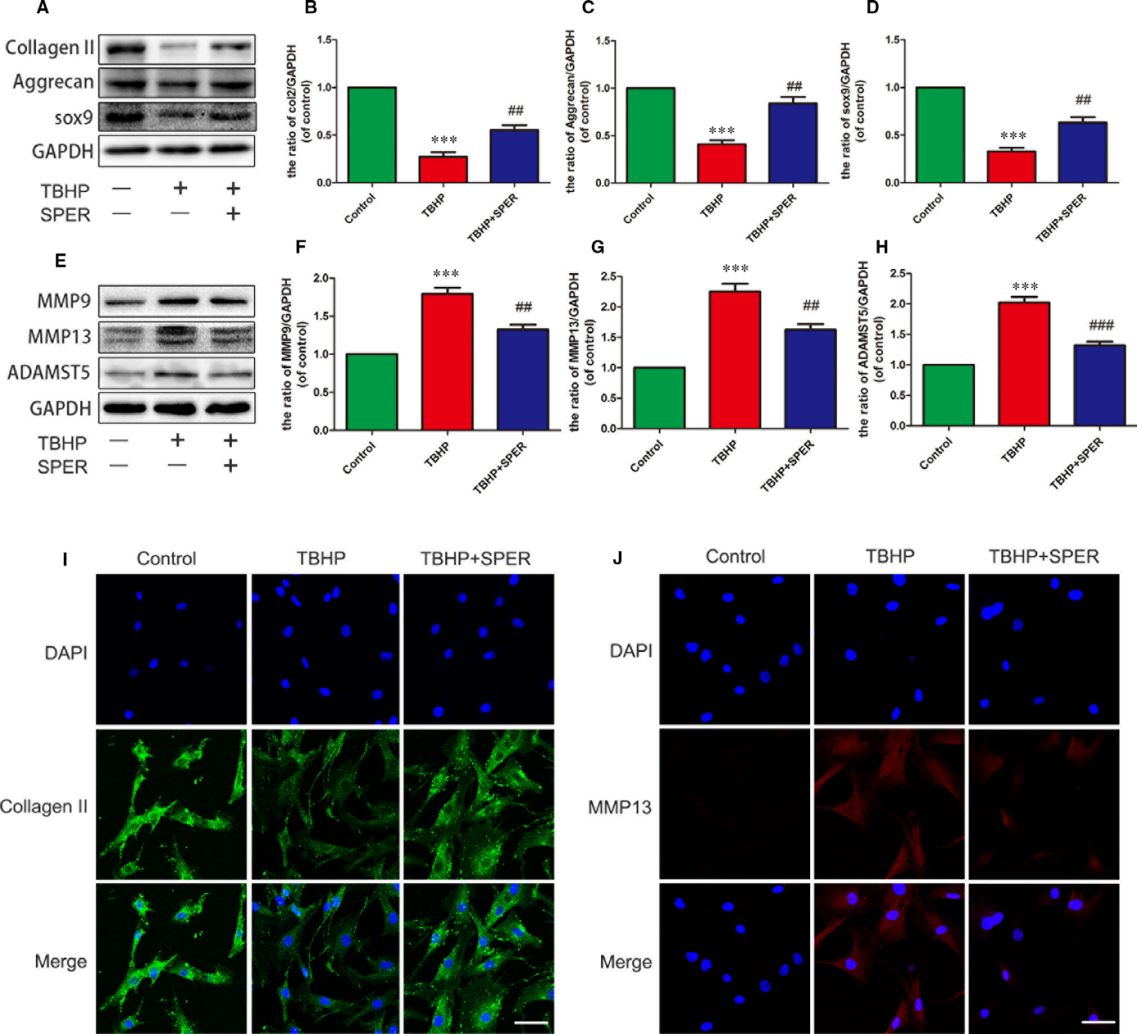
Spermidine regulates the expression of anabolic‐ and catabolic‐related proteins. Nucleus pulposus cells were untreated (DMEM 10%FBS), or treated with TBHP alone, or treated with spermidine (50 μmol/L) and TBHP, or treated with TBHP (A‐D) Protein content of Collagen‐II, Aggrecan, Sox‐9 of nucleus pulposus cells as treated above. (E‐H) Protein content of MMP9, MMP13, Adamts‐5 of nucleus pulposus cells as treated above. (I‐J)The representative Collagen‐II and MMP13 were detected by the immunofluorescence combined with DAPI staining for nuclei (Collagen‐II: original magnification ×400, scale bar: 25 μm, MMP13: ×400, scale bar: 25 μm). The data in the figures represent the averages ± SD. ***P < .001 vs the control group. ^##^ *P* < .01, ^###^ *P* < .001 vs TBHP group, n = 3 per group

To better evaluate the degeneration of NP cells, we investigated major ECM synthesis proteins, including Collagen‐II, aggrecan and sox‐9 as well major ECM degrading proteins, including MMP9, MMP13 and Adamts‐5. As shown in Figure 2A‐H, TBHP treatment was found to significantly reduce Collagen‐II, aggrecan and sox‐9 levels, and increase MMP9, MMP13 and Adamts‐5 protein expression levels (*P* < .01). Spermidine could reverse these TBHP‐induced effects. Spermidine treatment was found to increase Collagen‐II, aggrecan and sox‐9 protein levels and reduce the expression of ECM degrading proteins. Immunofluorescence analysis of Collagen‐II and MMP13 protein expression was consistent with Western blot results (Figure 2I,J). These results showed that spermidine could increase the expression of ECM synthesis proteins and reduce the expression of ECM degrading proteins.

### 3.3 Regulation of autophagy in nucleus pulposus cells cotreated with TBHP and spermidine

**Figure 3:**
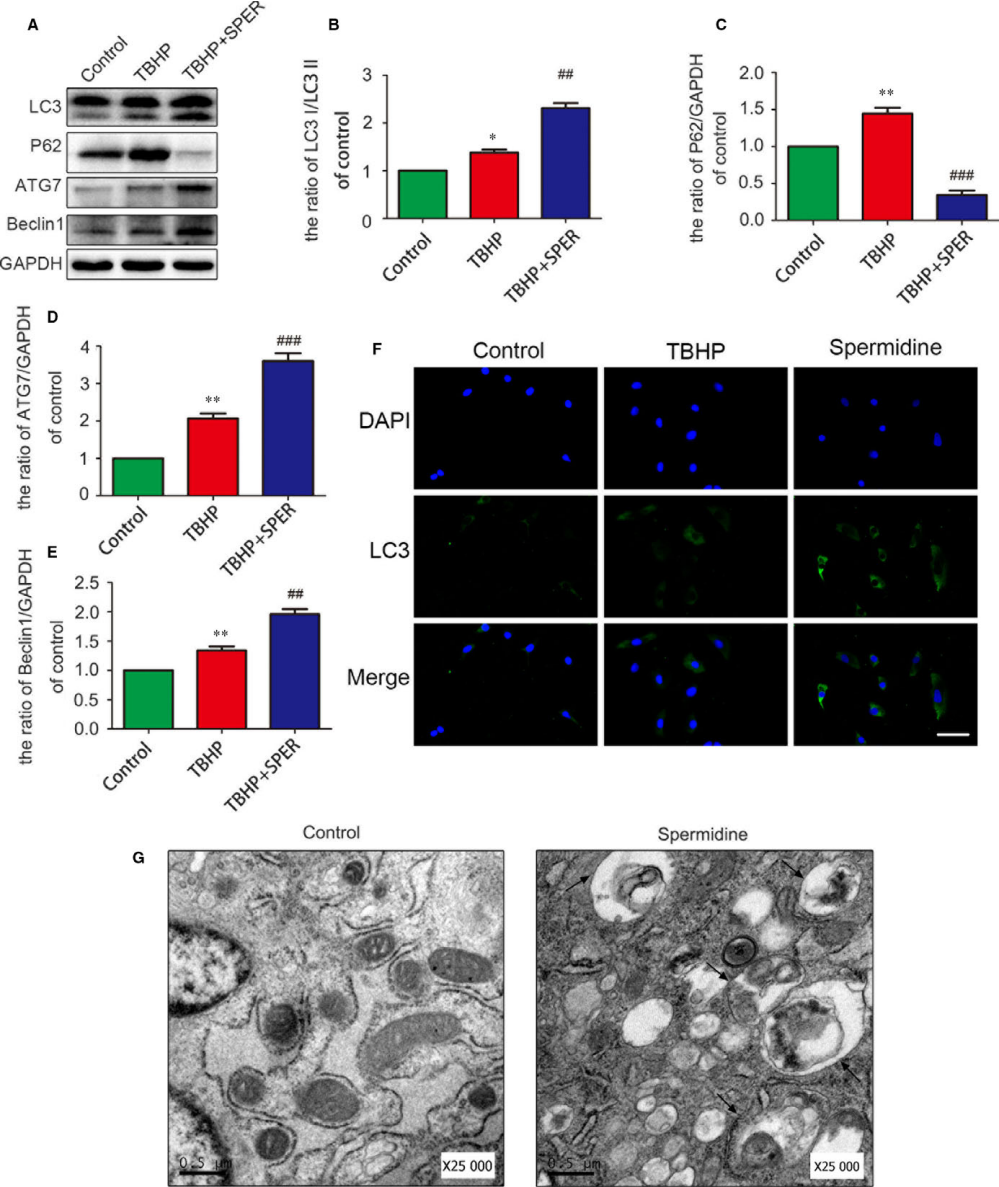
Regulation of autophagy in the nucleus pulposus cells cotreated with TBHP and spermidine. Nucleus pulposus cells were untreated, or treated with TBHP alone, or treated with spermidine (50 μmol/L) and TBHP, or treated with TBHP and spermidine. (A‐E) Protein content of Lc3, P62, Atg‐7, Beclin1 of nucleus pulposus cells as treated above. (F) Immunofluorescence of LC3 protein in nucleus pulposus cells. (Green signal represents LC3, scale bar: 25 μm). (G) Autophagosomes were detected by transmission electron microscopy (×25 000) in nucleus pulposus cells. (Black arrow: autophagosome). *P < .05, **P < .01 vs the control group. ^##^ *P* < .01, ^###^ *P* < .001 vs TBHP group, n = 3 per group

LC3‐II/LC3‐I ratio, Beclin‐1 and p62 were used as markers of autophagy induction. As shown in Figure 3A‐E, the ratio of LC3‐II/LC3‐I and the expression of Beclin‐1 in NP cells increased 24 hours after spermidine and TBHP cotreatment compared to the TBHP‐treated group. However, p62 level was found to be decreased under spermidine treatment compared with the TBHP group. Autophagosomes were visualized by transmission electron microscopy (Figure 3G). This is commonly used to assess autophagy activation. Compared to controls, the spermidine‐treated cells had an increased number of autophagosomes in their cytoplasm. These findings were corroborated by immunofluorescence staining for LC3, which revealed increased LC3‐II. Spermidine treatment increased LC3‐II immunoreactivity compared to TBHP‐treated cells (Figure 3F; *P* < .01). Thus, spermidine can markedly initiate autophagic flux in TBHP‐treated nucleus pulposus cells.

### 3.4 The protective effect of spermidine in NP cells is related to the stimulation of autophagy

**Figure 4:**
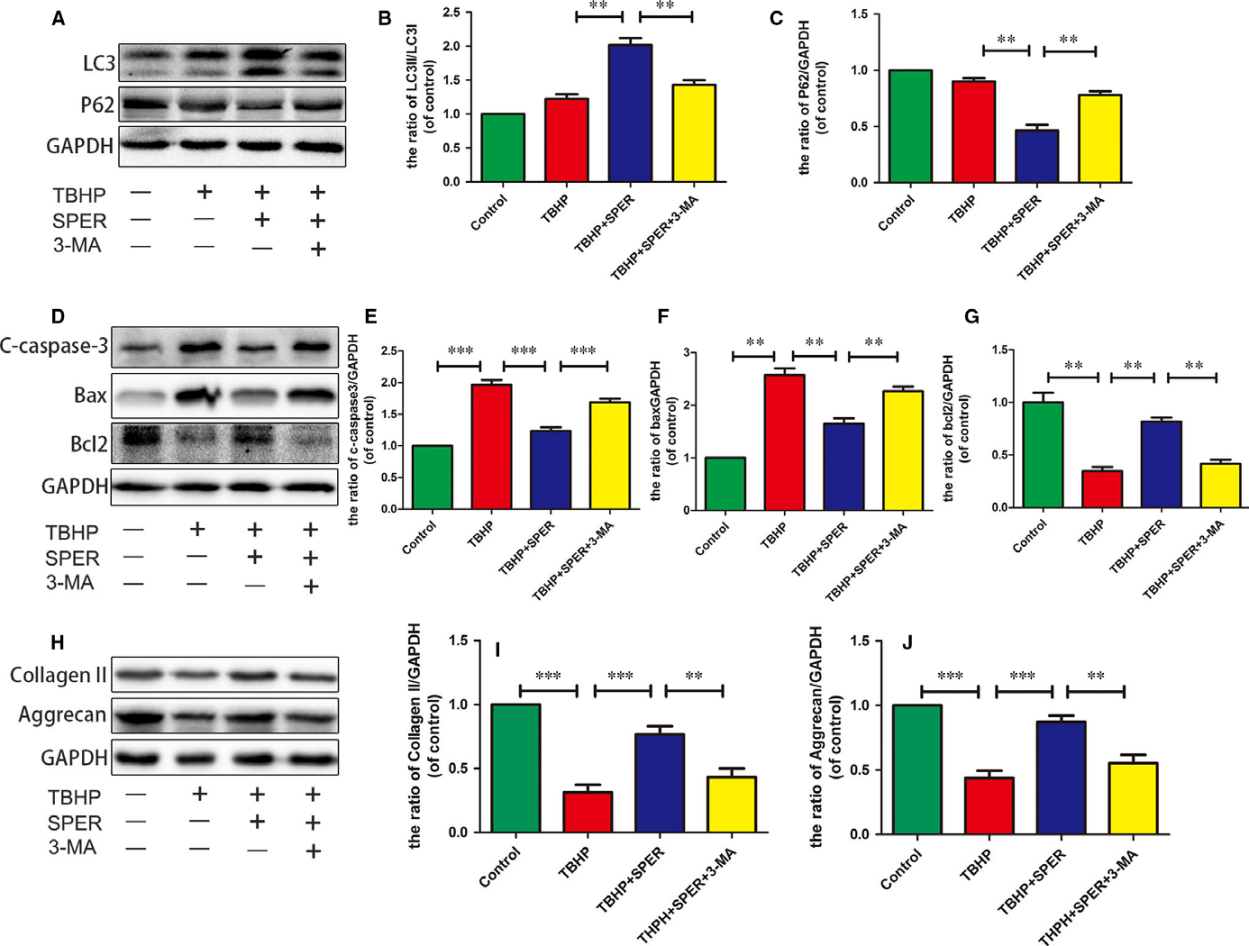
The protective effect of spermidine against apoptosis is related to the stimulation of autophagy. Nucleus pulposus cells were untreated, or treated with TBHP alone, or treated with spermidine (50 μmol/L) and TBHP, or treated with TBHP and spermidine (50 μmol/L) combined with 3‐MA (10 mmol/L). (A‐C) The protein expression of LC3, P62 in the nucleus pulposus cells as treated above. (D‐G)The protein expression of C‐casepase3, Bax, Bcl‐2 in the nucleus pulposus cells as treated above. (H‐J) The protein expression of Collagen‐II and aggrecan in the nucleus pulposus cells as treated above. The data in the figures represent the averages ± SD. Significant differences between the treatment and control groups are indicated as **P < .01, ***P < .001, n = 3 per group

To investigate whether autophagy was involved in spermidine's protective effect in NP cells, cells were pre‐treated with the autophagy blocker 3‐MA. As shown in Figure 4A‐C, 3‐MA treatment decreased the levels of LC3‐II/LC3‐I and increased p62 levels. In the spermidine group, the decreased the protein levels of Bax and cleaved caspase‐3 and the increased Bcl‐2 were reversed by 3‐MA treatment (Figure 4D‐G). We also tested the relationship between the matrix synthesis and degradation with autophagy. As shown in Figure 4H‐J, spermidine was promoted the synthesis of ECM, including Collagen‐II and aggrecan, when autophagy was inhibited by 3‐MA, the expression of Collagen‐II and aggrecan was down‐regulated. These results indicate that spermidine protects NP from oxidative stress induced by TBHP through autophagy.

### 3.5 Spermidine ameliorates rats intervertebral disc degeneration in vivo

**Figure 5:**
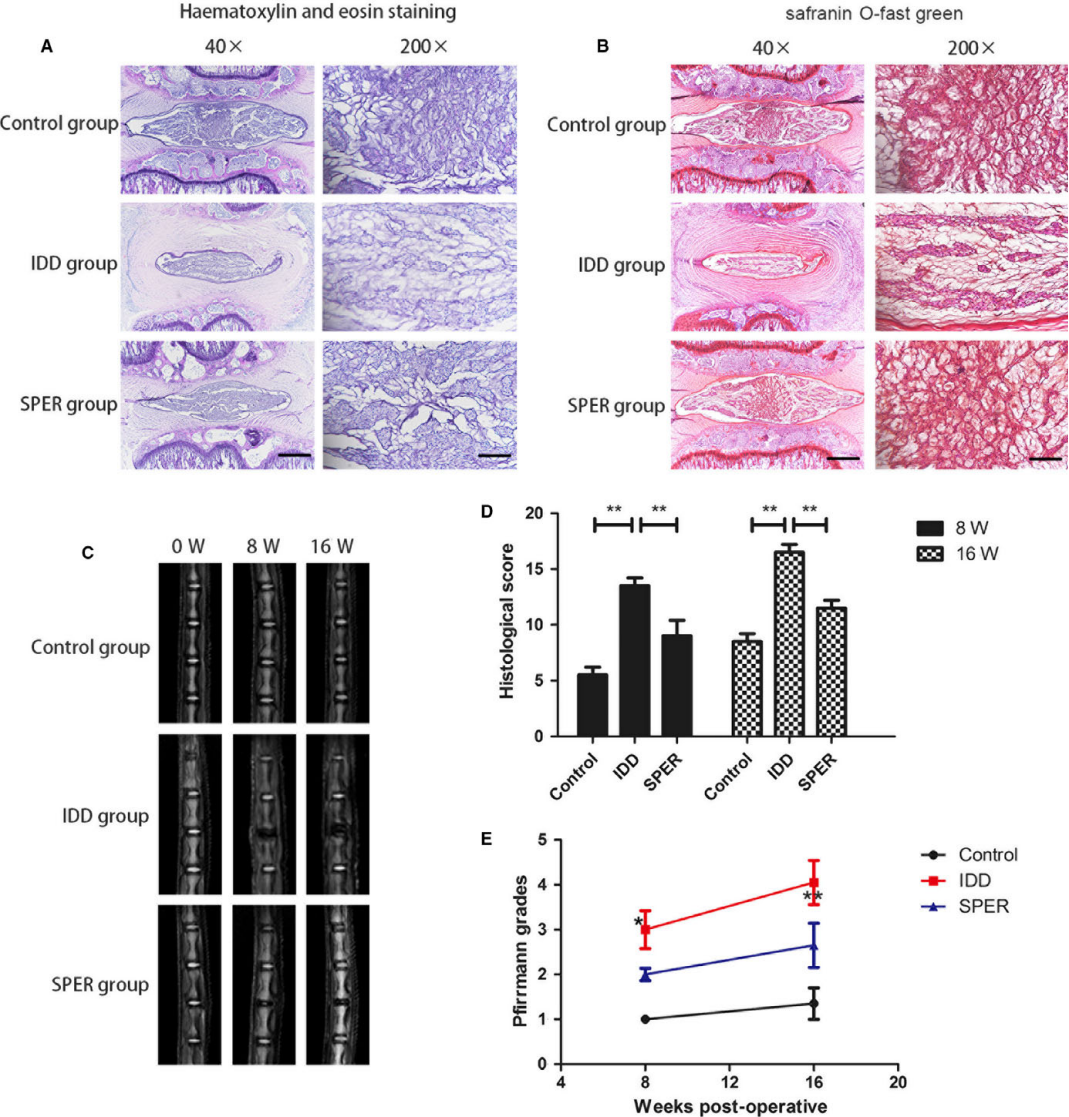
Spermidine treatment ameliorates rat IDD in vivo. (A‐B) Representative safranin O‐fast green and haematoxylin, and haematoxylin and eosin staining of disc samples from different experimental groups at 4 and 8 weeks post‐surgery. Scale bars are 200 μm (40×) and 50 μm (200×), respectively. (C) T2‐weighted MRI of a rat tail with a needle‐punctured disc at 4 and 8 weeks post‐surgery. (D) The histological grades evaluated at week 4 and week 8 in three groups. Samples from 48 rats (sixteen in each group) were used for imageological and histopathologic analysis. (E) The Pfirrmann MRI grade scores in three groups at week 4 and week 8. Significant differences between the treatment and control groups are indicated as **P < .01, *P < .05, n = 6 per group

The beneficial effects of spermidine against IDD were examined by histological staining at 4 and 8 weeks in the rat degenerative model. (Figure 5A,B) Nuclear cells in control discs were evenly distributed and stellar‐shaped in the NP, with the proteoglycan matrix arranged in slim lines. Compared to controls, the size of the NP in the saline group decreased progressively while the size of the nuclear cells increased and became more rounded. In addition, they categorized into clusters that were divided by compressed areas of proteoglycan matrix, which indicated severe degeneration of the NP cells. Spermidine treatment was found to significantly alleviate loss of nucleus pulposus tissue and destruction of disc structure compared with the IDD group. The histologic score of the spermidine group was markedly lower than that of the IDD group both at week 8 (*P* < .01) and week 16 (*P* < .01; Figure 5D). The different levels of IDD in rats were assessed by magnetic resonance imaging (MRI) and Pfirrmann MRI grade scores. MR images obtained at 8 weeks after puncture had stronger T2‐weighted signal intensities in the spermidine‐treated group than that in the IDD (saline) group. Similar results were also observed at 16 weeks (Figure 5C). In addition, Pfirrmann MRI grade scores, which indicate the degree of disc degeneration, were found to be significantly lower in the spermidine‐treated rats than that in the IDD (saline) group at 8 weeks (*P* < .05) and 16 weeks (*P* < .01; Figure 5E).

## 4 DISCUSSION

Intervertebral disc degeneration (IDD) has been the primary cause of lower back pain in recent years.^2^ With a rapidly ageing population, exploration for a new drug treatment promoting stimulation of endogenous repair and retarding IDD is vital. Our study demonstrated that spermidine treatment induces autophagy in NP cells, which blocked NP cell apoptosis and degenerate protein expression induced by oxidative stress. In addition, spermidine may play a protective role in IDD in a puncture‐induced rodent model.

Many studies have reported that the progression of IDD is closely associated with oxidative stress.^23^ Excessive reactive oxygen species (ROS) generation in degenerative intervertebral discs (IVD) supports this model; thus, providing an original perspective into IDD pathogenesis.^20^ ROS are crucial intermediators in the signalling network of disc cells, which could induce cell apoptosis through mitochondrial dysfunction^24^ and provoke premature senescence.^20^ In this study, we showed that the administration of TBHP increased NP cell apoptosis, which was significant to the pathogenesis of IVD degeneration.

Autophagy is an intracellular process used to recycle cytoplasmic components through lysosomal degradation to support cell homoeostasis.^25^ Research in various model organisms has found that autophagy is a vital mediator of the ageing process.^26^, ^27^ Autophagic decline can initiate neurodegenerative phenotypes in mice,^28^ and autophagy‐mediated clearance of TDP‐43‐positive inclusions can preserve learning difficulties associated with neurodegenerative diseases relating to TDP‐43 proteinopathies.^29^ Degenerated disc tissue has increased activation of autophagy‐related genes and proteins compared with healthy tissue.^30^ Autophagy initiation through the Ca2 ± ‐dependent AMPK/mTOR pathway may result from an adjustment system for notochordal cells experiencing hyperosmotic stress.^31^ Activating autophagy in disc cells may be an innovative therapeutic outlook in the pathogenesis of IDD. Rapamycin has been shown to be a powerful activator of autophagy. However, it has been to shown to have significant side‐effects, including hyperlipidemia, diabetes‐like symptoms and enhanced risk of infection.^32^ Therefore, it is clinically significant to screen for new drugs targeting autophagy activation with mild adverse side‐effects.

Spermidine is an autophagy‐mediating polyamine, and interest for its application in a clinical setting is a growing avenue of nutraceutical therapy.^33^ Recent research has found that spermidine is beneficial for countering a variety of age‐associated pathologies, including both memory impairment^17^ and arterial ageing.^34^ Spermidine has been found to counteract ageing and promote longevity through increased basal autophagy.^15^, ^35^, ^36^ Moreover, re‐activation of basal autophagy by spermidine has recently been reported to maintain the regenerative function of skeletal muscle stem cells in ageing mice.^27^ It has also been shown to induce autophagy in various tissues, including the heart,^16^ brain^17^ and cancer cells.^37^ In this study, we found that spermidine may activate autophagy in NP cells. Spermidine, a natural polyamine the intracellular level of which declines during ageing. It is safe in application to cell culture media or in vivo to animals. In our present study, we proved that spermidine treatment inhibits apoptosis and regulates matrix synthesis, spermidine alone group were tested, and we found that there is no significant difference with control group. This result is in line with the previous literature.^38^

It is widely known that apoptosis may be involved in IDD pathophysiology, which suggests that it is important for intervertebral disc degeneration.^39^ Extreme apoptosis has been found to reduce the activity of the nucleus pulposus and decrease extracellular matrix changes in synthesis and composition, which further contributes to IDD pathology.^40^ Mitochondrial dysfunction caused by oxidative stress, including decreased Bcl‐2 and release of Bax triggers the initiation of caspases leading to apoptosis.^41^, ^42^ In the present study, we found that pre‐treatment with spermidine could significantly decrease Bax and cleaved caspase‐3, while increasing Bcl‐2 in NP cells subjected to oxidative stress. This suggests that the mitochondrial pathway may mediate spermidine's anti‐apoptosic effect. Moreover, spermidine was promoted the synthesis of the most prominent components of ECM, including Collagen‐II and aggrecan and inhibited the ECM breakdown by down‐regulating the matrix‐degrading enzymes MMP13 and Adamts‐5, which help to maintain nucleus pulposus homoeostasis. In the experiments related to autophagy, we have tried spermidine alone and of 3‐MA alone group as control, in autophagy‐related proteins, the results of the spermidine alone group were not significantly different from those of the SPER+TBHP group, and 3‐MA alone group were similar to those of the control group. This result is consistent with the previous literature. Spermidine is known to promote cellular survival through autophagy.^43^ Spermidine induced autophagy and mitophagy in the heart and exerts its cardioprotective effect.^44^ 3‐MA, a known autophagy inhibitor, was applied in our study.^45^ We found that these beneficial effects were lost when the autophagy was inhibited using 3‐MA, when autophagy was inhibited, the apoptosis of nucleus pulposus increases, simultaneously, components of ECM, including Collagen‐II and aggrecan decrease; therefore, autophagy inhibition not only affects the apoptosis of NP cells, but also inhibits the synthesis of extracellular matrix. These results indicating that the protective effects of spermidine were mediated by autophagy stimulation.

MMPs are essential in the catabolism of ECM in intervertebral disc degeneration,^46^, ^47^, ^48^ as well as Adamts‐5, which are mainly responsible for the degradation of aggrecan. Studies have reported that oxidative stress could regulate the expression of MMPs or Adamts in the pathogenesis of IDD and other degenerative diseases.^49^, ^50^, ^51^ MMP or ADAMTS can serve as a target for the treatment of IDD. The non‐coding RNA linc‐Adamts‐5, which blocked Adamts‐5 expression, was protective against IDD.^52^ Adamts‐5 siRNA could also effectively suppress Adamts‐5 production in the nucleus pulposus, improving both imaging and histologic grade in a rabbit needle‐puncture model.^53^ We demonstrated that TBHP significantly increases MMP13 and Adamts‐5 expression. However, spermidine supplementation resulted in decreased in MMP13 and Adamts‐5 protein. Furthermore, it attenuated the matrix loss of Collagen‐II and aggrecan in NP cells. Simultaneously, spermidine treatment retarded T2‐weighted signal intensities in puncture‐induced IDD rats. Therefore, these results may indicate that spermidine treatment protects against the matrix loss induced by TBHP through decreased expression of ECM catabolic enzymes.

In conclusion, this study suggests that treatment with spermidine induces autophagy that may be protective against apoptosis in NP cells. Interestingly, it exerts anti‐apoptosic effects against oxidative stress, whereas autophagy blockade eliminates these effects. These findings indicate a therapeutic potential for spermidine in mitigating disc degeneration. In light of this evidence, spermidine may be successfully applied to IDD therapy. This could be further extrapolated to include a range of inherited musculoskeletal diseases in which defective autophagy machinery is commonplace.

## CONFLICT OF INTEREST

The authors declare that they have no conflict of interest.
